# Report of Diseases in the Public and Private Practice of One of the Physicians of the Finsbury Dispensary, from the 20th of July to the 20th of August

**Published:** 1805-09-01

**Authors:** J. Reid

**Affiliations:** Grenville Street, Brunswick Square


*Report of Diseases in the public and private Practice of one of the Physicians of the Finsbury Dispensary, from the 20th of July to the 20th of August.*

| | | | |
|---|---|---|---|
| Febris | 2 | Hypochondriasis | 9 |
| Cholera | 1 | Epilepsia | 1 |
| Diarrhœa | 17 | Amenorrhœa | 14 |
| Rheumatismus | 3 | Cephalalgia | 1 |
| Phthisis | 8 | Anasarca | 5 |
| Catarrhus | 8 | Morbi Cutanei | 16 |
| Dyspnœa et Tapis | 13 | Morbi Infantiles | 19 |
| Dyspepsia | 12 | | |

The last month has not been marked by the extraordinary predominance of any individual disease. Complaints of the bowels have indeed, as is usual about this period of the year, prevailed to a considerable extent; but decided cholera has been rare.

A remarkable instance has recently occurred, in which a fit of epilepsy almost immediately followed a paroxysm of anger. An attack of what are called nervous affections, in all their various and miscellaneous forms, not unfrequently originate from some agitation or impetuous move-

 ment ment of the mind. The important influence of ill-managed passions is by no means sufficiently appreciated.

To a careless adjustment or an insufficient regulation of the mental, are to be attributed, much more frequently than it is in general imagined, the disorders and anomalous irregularities that occur in the corporeal department of our frame.

Pharmacy is but a small part of physic. In the successful treatment of disease, other and more powerful agents must often be employed than are to be found amidst the medicinal variety of the shops. The art of *healing* implies, in a metaphorical as well as a literal sense, a knowledge of the human heart—the anatomy of the mind as well as that of the body. Medical cannot be separated from moral science, without reciprocal and essential mutilation.

This remark applies more particularly to a proper knowledge and treatment of their complaints, whose rank and circumstances in life entitle them to the falsely envied privileges of luxury and leisure.

The diseases of the poor and the rich are not essentially different. Similar debility and disorders are produced in the one instance directly, and in the other indirectly, by a very full and high, or by a very low and meagre regimen. The indigent wretch, whose scanty fare scarcely is sufficient to support the stamina of existence, and the no less wretched debauchee, whose intemperate indulgence daily accelerates the period of his destruction, may both with an equal propriety be said to *live hard*. The only important distinction that exists between the diseases of the vulgar and of the more fashionable world, arises from the former being so entirely engrossed in supplying the necessities of life, and in suffering from its physical inconveniencies, as not to be at sufficient liberty to feel and contemplate those infinitely more dreadful calamities that grow out of the soil of a pampered and consequently distempered imagination. A person must be idle in order to be perfectly miserable. No evil is worse than that intolerable sense of vacuum which the mind suffers that has no object commensurate to its capacity, or whose faculties of action and of feeling, although in a state of requisition, are not summoned by an imperious necessity, or other motives of sufficient power, to regular and interesting occupation. To the proper and healthy state of man daily exertion is no less necessary, than the diurnal motion of the earth he inhabits is to its existence and continued preservation.

servation. Without intellectual, bodily exercise is comparatively of little avail to one whose understanding has been enriched and exalted by literary cultivation. "I will not hesitate to assert, that to have the mind ardently engaged in a pursuit that totally excludes exercise of the body, is much more favourable to the spirits than a languid mixture of both."* Of the important effects arising from bodily labour, when united with mental excitement, we have recorded a remarkable instance in the "Monita et Precepta" of Dr. Mead.—"A young student at college became so deeply hypochondriac, that he proclaimed himself dead, and ordered the college-bell to be tolled on the occasion of his death. In this he was indulged; but the man employed to execute the task appeared to the student to perform it so imperfectly, that he arose from his bed in a fury of passion to toll the bell for his own departure. When he had finished, he retired to his bed in a state of profuse perspiration, and was from that moment alive and well.—"*Vitam autem reddidit iste labor, et convalescit.*"

J. REID.

*Grenville Street, Brunswick Square, August* 25, 1805.

* Dr. Aikin's "Letters to his Son."

